# Optimal utilisation of sulphonylureas in resource-constrained settings

**DOI:** 10.5830/CVJA-2014-007

**Published:** 2014-04

**Authors:** Poobalan Naidoo, Virendra Rambiritch, Neil Butkow, Selvarajah Saman

**Affiliations:** Boehringer Ingelheim, Johannesburg, South Africa; University of Kwa-Zulu Natal, Durban, South Africa; Department of Pharmacy and Pharmacology, Faculty of Health Sciences, University of Witwatersrand, Johannesburg, South Africa; Port Shepstone Regional Hospital and University of Kwa-Zulu Natal, Durban, South Africa

**Keywords:** type 2 diabetes mellitus, sulphonylureas, resource-constrained settings

## Abstract

**Abstract:**

Sulphonylureas (SUs) are oral anti-diabetic drugs (OADs) that were introduced more than 60 years ago. Clinicians are familiar with their use and they remain extensively used. However, the SU class is associated with adverse effects of weight gain and hypoglycaemia. In addition, their effects on cardiovascular events remain contentious. Newer classes of anti-diabetic agents have been developed and these agents are weight neutral (di-peptidyl peptidase IV inhibitors), while others reduce weight (glucagon-like peptide analogues and sodium glucose co-transporter inhibitors). Furthermore, the newer agents are less likely to cause hypoglycaemia and have a potentially better cardiovascular safety profile. However, the newer agents are more costly than SUs and their long-term safety is unknown. It is therefore likely that SUs will continue to be used, and more so in resource-limited settings. One may mitigate the adverse effects of weight gain and hypoglycaemia associated with the SU class by using members within this class that are less probable to cause these adverse effects. Furthermore, the specific SU must be used at the lowest effective therapeutic dose. In patients at high risk of SU-induced hypoglycaemic episodes (frail, clinically significant renal impairment), or patients in whom hypoglycaemic episodes may have devastating effects (bus drivers), newer anti-diabetic agents may be a justifiable alternative option.

## Abstract

Sulphonylureas (SUs) were developed in the 1950s.1 They reduce blood glucose levels by increasing insulin secretion from the pancreatic beta-cells. At the cellular level SUs block potasssium (KATP) channels and increase calcium influx, which results in the release of insulin from the vesicles.^1^

Currently there is an expansion in the therapeutic armamentarium of agents for type 2 diabetes. The therapeutic landscape is complex and comprises pharmacologically distinct molecules, including biguanides, sulphonylureas, incretin-based therapies and renal sodium glucose co-transporter (SGLT) inhibitors.^2^ As novel therapies are inevitably associated with increased costs, this article focuses on ways to utilise SUs in a manner that maximises efficacy and concurrently minimises adverse effects.

## Efficacy and durability of glycaemic effect

Type 2 diabetes patients benefit from intensive multifactorial riskfactor modification.^3^ In addition to control of blood glucose and glycosylated haemoglobin (HbA_1c_) levels, lifestyle modification (diet and exercise), and control of blood pressure and cholesterol levels are crucial to reduce the risk of cardiovascular disease in type 2 diabetes patients.^3^

For blood glucose control, HbA_1c_ level is the most robust endpoint used in clinical trials to evaluate the efficacy of antidiabetic drugs. HbA_1c_ is an indicator of three-month average blood glucose levels. Reduction in HbA1c levels reduces microvascular complications.^4^-^6^

SUs reduce HbA_1c_ levels by approximately 1.5%,^2^ but their effect on cardiovascular outcomes is contentious. Their HbA_1c_ level-reducing ability is adequate but durability is limited.^7^ Limited durability is probably secondary to type 2 diabetes mellitus being a progressive disease characterised by gradual reduction in beta-cell mass and function. If there are limited numbers of beta-cells, then the action of this class is limited because the mode of action necessitates the presence of betacells; they cannot increase insulin secretion if there are no betacells present to synthesise and release insulin.

Furthermore, secondary failure has also been attributed to the detrimental effects of SUs on residual pancreatic beta-cells.^8^ Secondary failure rates were found to be lowest with gliclazide (7%), compared with glibenclamide (17.9%) and glipizide (25.6%).^9^

## Safety data

SUs cause weight gain^2^,^10^ and significantly increase the risk of hypoglycaemia.^11^,^12^ Hypoglycaemia appears to be associated with adverse vascular events and death.^13^

There are also issues with regard to cardiovascular safety. There is inconsistency in the results of clinical studies in respect of SUs and cardiovascular safety. The University Group Diabetes Program^14^ demonstrated increased cardiovascular mortality in patients treated with tolbutamide. However, the United Kingdom Prospective Diabetes Study (UKPDS)^4^ and the ADVANCE Collaborative Group^5^ did not show an association between treatment with an SU and adverse cardiovascular outcomes.

In a meta-analysis of 33 studies, with more than a million study subjects, SU use was associated with a significantly increased risk of cardiovascular death (relative risk 1.27, 95% confidence interval 1.18–1.34, *n* = 27 comparisons).^15^ Monami *et al.*^16^ conducted a meta-analysis of randomised clinical trials to evaluate the cardiovascular safety of SUs. They concluded that ‘in type 2 diabetes, the use of sulfonylureas is associated with increased mortality and a higher risk of stroke, whereas the overall incidence of major adverse cardiovascular events (MACE) appears to be unaffected’.

Given the inconsistency of the literature with regard to SUs and cardiovascular outcomes, a SU cardiovascular outcome trial is required to clarify the effect of SUs on cardiovascular outcomes.^16^,^17^

## Dose–response relationships

The literature supports the use of SUs at doses lower than the maximum manufacturer’s recommended dose.^18^ Studies have shown that as the dose of SU is increased, there is initially a direct relationship between dose and blood glucose-lowering effect.^18^ However, further dose increase results in no further reduction in blood glucose levels, and, when the dose is further increased, the glycaemic profile actually worsens.^18^

Modified-release formulations have further reduced the SU dose that is required, compared to the immediate-release pharmaceutical preparation.^19^ For example gliclazide is available in a modified-release formulation that uses less than half of the dose of the immediate-release formulation.^19^

## Cost considerations

SUs remain affordable. This is relevant in countries that have limited resources and competing healthcare problems. In sub-Saharan Africa, there are epidemics of not just metabolic and cardiovascular disease, but also infectious diseases.^20^ Tuberculosis and parasitic diseases such as malaria remain major healthcare challenges, while diabetes, hypertension and traumatic injuries are increasing.^21^ Therefore scarce medical resources must be distributed to various disease-management programmes.

However, one may argue that managing SU-induced hypoglycaemic events (the cost of treating and in some cases the cost of admission), raises their cost. One may mitigate this added cost by using the newer SUs that have fewer propensities to cause hypoglycaemia compared to older agents.

## Newer classes of anti-diabetic agents

The ideal anti-diabetic drug should be safe, efficacious and cost effective. It should not only reduce HbA1c levels but also reduce macro- and microvascular complications. Furthermore, it must not cause weight gain and hypoglycaemia, and must have durable efficacy and long-term safety. There is continuing research to develop newer agents to emulate the characteristics of an ideal anti-diabetic agent, and therefore better manage type 2 diabetes patients.

Sodium glucose co-transporter (SGLT) inhibitors and incretin-based therapies are new classes of anti-diabetic agents. SGLT inhibitors reduce weight and have fewer propensities to cause hypoglycaemic events.^22^ This is in contrast to the SU class that increases weight and the number of hypoglycaemic episodes.

Incretin-based therapies include glucagon-like peptide (GLP) analogues and di-peptidyl dipeptidase IV (DPPIV) inhibitors. GLP analogues reduce weight but are administered via the parenteral route. DPPIV inhibitors are weight neutral, have a low propensity for hypoglycaemia and are administered orally.

The uncertainty surrounding adverse cardiovascular events associated with therapy with SUs remains,^15^ in contrast to the DPPIV class, which has both meta-analysis^23^ and a cardiovascular outcome trial^24^ that demonstrate cardiovascular safety of this new class.

There are safety concerns with newer anti-diabetic agents. For example, issues related to pancreatitis and pancreatic cancer remain with incretin-based therapies.^25^ However, the American Diabetes Association (ADA), European Association for the Study of Diabetes (EASD) and the International Diabetes Federation (IDF) have issued a joint statement saying that there is inadequate information presently to demonstrate a causal relationship between incretin-based therapy and pancreatitis and pancreatic cancer.^26^ There are also concerns with the SGLT inhibitor class and bladder and breast malignancies, and urinary and genital tract infections.^22^ The newer agents require further phase IV data to inform clinical use.

## Maximising benefits and minimising adverse effects of SUs

After considering the adverse effects, safety concerns, efficacy data and cost, one must use SUs in a manner that maximises efficacy while limiting the potential for adverse effects. The question is how does the clinician do this? One way is to choose the ‘right sulphonylurea, at the right dose, for the right patient’.

The right sulphonyureas: the SUs share a common mode of action. However, there are differences in pharmacokinetics and pharmacodynamics between individual SUs. Some SUs have fewer propensities for hypoglycaemia and weight gain than others.^27^

South African treatment guidelines for type 2 diabetes specifically mention that glibenclamide must be phased out, and in the interim it must be dispensed only if renal function is known.^28^ Data derived from the UK General Practice Research Database (719 general practitioner practices, 34 052 patient-years of SU therapy) reported that in users of SUs, the annual risk of any hypoglycaemic event was 1.8%, rising to 2.0% in those aged > 65 years. The risk of SUs was greatest for glibenclamide; the study reported 25% fewer recorded episodes for gliclazide and 40% fewer for glipizide compared with glibenclamide.^29^

At the right dose: given the data on dose–response relationships of the class, it is prudent to use the lowest effective dose of SU, guided by efficacy parameters such as HbA_1c_ levels.

For the right patient: SUs are more likely to cause adverse effects in patients with risk factors for hypoglycaemia, including older, frail patients and patients with clinically significant renal impairment.^30^ In addition, any hypoglycaemic effect may be devastating for specific patients, such as bus drivers. Therefore SUs should perhaps be avoided in these groups and newer antidiabetic drugs considered.

## Conclusion

Cost issues remain a barrier between the newer anti-diabetic drugs and the majority of South African type 2 diabetes patients. SUs, if used at the right dose (the lowest possible effective dose), for the right patient (in younger patients without renal impairment), remain an option for the management of type 2 diabetes patients in resource-constrained settings.
